# Left Ventricle Outflow Obstruction by Reverse-Oriented Tricuspid Semilunar Valve-Like Endocardial Duplicatures

**DOI:** 10.1155/2018/2403806

**Published:** 2018-05-08

**Authors:** Kristína Mikuš-Kuracinová, Pavel Babál, Eliška Kubíková

**Affiliations:** Department of Pathology and Institute of Anatomy, Comenius University Faculty of Medicine Bratislava, Sasinkova 4, 81372 Bratislava, Slovakia

## Abstract

A 57-year-old female had a history of hypertension disease, and one year before her death, her ECG showed signs of left ventricle hypertrophy. She died with signs of heart failure with pulmonary edema development. At autopsy, there was left ventricle hypertrophy (wall thickness: 21 mm). In the left ventricle outflow channel, 15 mm below the aortic valve on the muscular wall, there were three white 1–1.5 mm thick membranous semilunar valve-like structures with the sizes of 9, 7, and 5 mm, with concavities opened into the left ventricle, reducing the outflow area by 21.5%. These structures were hanging on the regular muscular ventricular wall, without any visible fibrous anchoring structure and without formation of commissures, and were composed of fine collagen and elastic fibers. Gross anatomy as well as histological structure was different from the subaortic membrane. The reported accessory reverse-oriented tricuspid semilunar valve-like structure is an unusual finding of a structure in the left ventricular outflow tract, to which we could not find an analogy in the available literature.

## 1. Introduction

The heart is a three-dimensional organ with complicated configuration including the four heart valves pushing the blood to flow in a unidirectional way [1]. Defects of the heart valves represent the most frequent congenital pathological condition in the heart [2–5].

Subvalvar aortic stenosis corresponds to about 1% of congenital heart diseases. A rare cause of this congenital condition is the obstruction by an accessory mitral valve tissue, approximately half of these corresponding to an accessory mitral leaflet [6, 7]. Discrete subvalvar aortic stenosis represents a unique cardiac lesion. As compared to most other congenital heart defects, the last mentioned is virtually never recognized in early infancy but appears to be an “acquired” lesion, although with anatomic precursors [8]. The stenosis is caused by a fibrous ridge in the left ventricular outflow tract just proximal to the aortic valve [9]. Here, we report an unusual bizarre finding of an accessory reverse-oriented tricuspid semilunar valve-like structure in the left ventricular outflow, found postmortem.

## 2. Case

A 57-year-old woman was admitted to hospital for ileus status and metabolic disturbance. She had a history of paranoid schizophrenia, thyroid gland hypofunction, and hypertensive disease. No records of positive heart auscultation finding were found. Her ECG 1 year before death showed signs of left ventricular hypertrophy; echocardiography was not performed. The patient died with symptoms of heart failure and was indicated for autopsy. At autopsy, the main findings were distended intestines with numerous *skybala* in the colon and pulmonary edema.

## 3. The Heart Gross Finding

The heart was 10 × 12 × 7 cm; the ventricles were in diastolic position, left ventricle wall 21 mm, right ventricle 4 mm thick, myocardium on cross-section was brown, with a white hard elastic focal lesion of 10 mm in diameter in the middle of the anterior wall of the left ventricle; circumference of the tricuspid valve was 13 cm, mitral valve 10 cm, pulmonary valve 8 cm, aortic valve 7 cm; the valves were anatomically correct, leaflets were elastic, without thickening. The coronary arteries were without significant narrowing, with fibrous plaques covering 10% of the intima.

**Figure 1:**
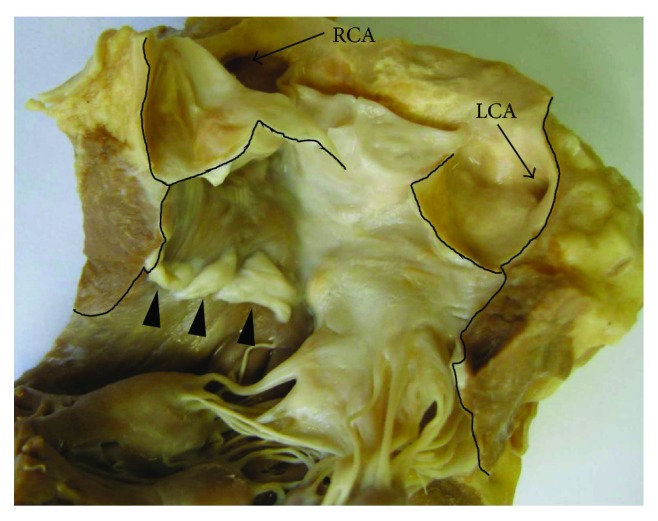
Left ventricle outflow channel opened with the cut delineated by the black line. On the ventricular septum wall, there are three membranous semilunar valve-like structures with concavities opened into the ventricle (arrowheads). Coronary arteries' ostia—left: LCA; right: RCA.

In the left ventricle outflow channel, 15 mm below the aortic valve on the muscular wall, there were three white 1–1.5 mm thick membranous semilunar valve-like structures with the sizes of 9, 7, and 5 mm, with concavities opened into the left ventricle. These structures were hanging on regular muscular ventricular wall, without any visible fibrous anchoring structure, and without formation of commissures (Figure 1). The internal area of the left ventricle outflow was reduced 21.5% by the valve-like structures.

### 3.1. The Heart Histology

**Figure 2:**
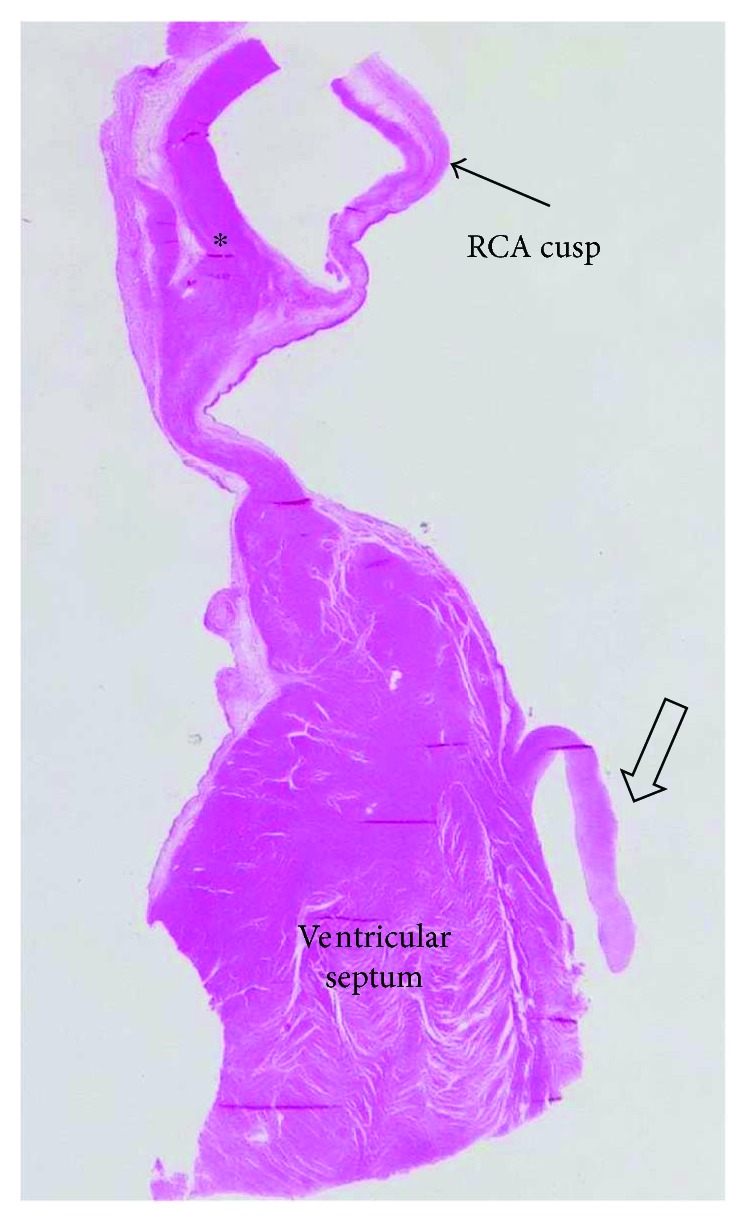
Section through the muscular wall of the left ventricular outflow and the aortic root. Membranous valve-like structure (large arrow) on the ventricular septum, aortic wall (asterisk), and the right coronary artery cusp (RCA cusp) of the aortic valve.

In the myocardium, there was diffuse interstitial fibrosis and myocytes were enlarged, with large hyperchromic nuclei. The endocardium was without any significant thickening. Direct projection of the histological slide shows the valve-like structure hanging on the ventricular wall with normal myocardium appearance, without fibrous anchoring structure unlike the normal aortic valve (Figure 2).

**Figure 3:**
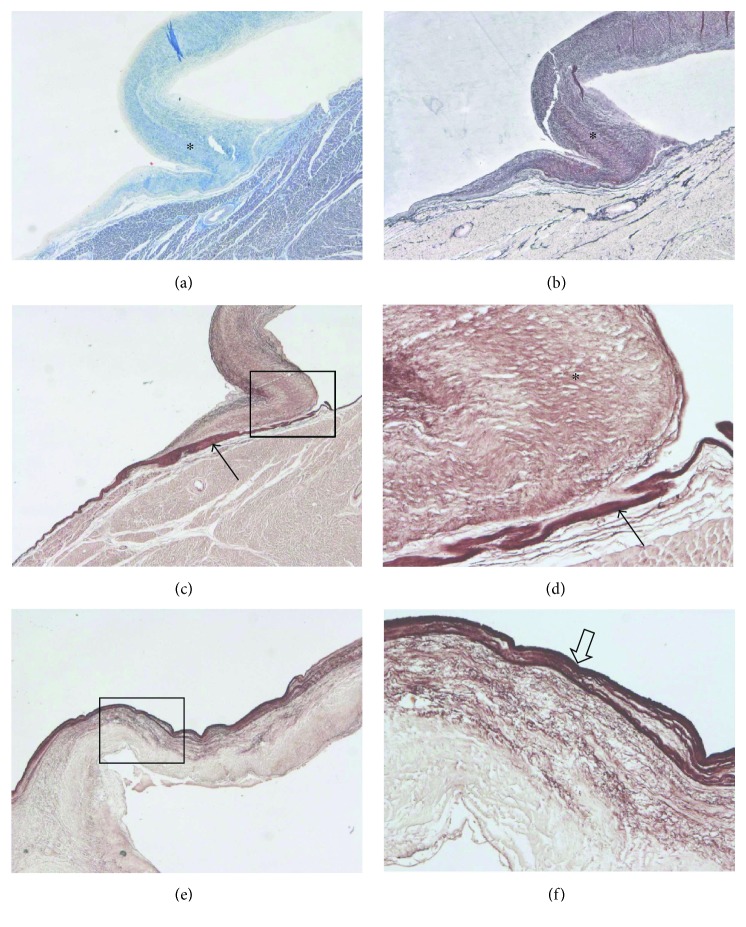
The valve-like structure was composed of fibrillary material with staining properties of fine fibrillary collagen (asterisk) stained with trichrome (a) and methenamine silver impregnation (b), with scattered very fine elastic fibers stained with orcein (c, d); the anchorage of the valve-like structure was on a thickening of the adjacent endocardium with a coarse compact layer of elastic fibers (arrow, c, d). Normal aortic valve leaflet is underlayed on the ventricular aspect by a continuous thick layer of elastic fibers (e, f, large arrow) separating into thinner subunits (orcein). 25x (a, b, c, e). 100x (d, f).

The valve-like structures were composed of fibrillary material with staining properties of collagen with scattered very fine elastic fibers, only lightly more condensed on both surfaces. There was no evident continuation or anchorage of fiber fascicles of the leaflets into the left ventricle wall. The only finding was thickening of the adjacent endocardium with a coarse compact layer of elastic fibers (Figure 3). This was different from the normal aortic valve leaflet, which was underlayed on the ventricular aspect by a continuous thick layer of elastic fibers that had a tendency to separate into thinner subunits towards the edge of the leaflet (Figure 3(f)). The normal aortic valve leaflet was outgoing from structures of the annulus fibrosus formed by dense meshwork of fine collagen fibers (Figure 2).

## 4. Discussion

Most frequent pathologies affecting the left ventricular outflow are inborn defects of the aortic valve. Embryologically, the semilunar valves are derived from mesenchymal swelling in the aortic and pulmonary trunk after they have been separated. Abnormal cusp formation results either from aberrant fusion of the aorticopulmonary septum or from abnormal mesenchymal proliferation in the common trunk, clinically leading to a severe regurgitation [4, 10]. The abovementioned inborn defects of aortic valve development encompass the tissues of the valve.

The substantial difference of the valve-like structures found in our case differed from normal aortic valve cusps in their histological structure. No collagen accumulation in the left ventricular outflow wall was observed that could be considered an analogy of the annulus fibrosus [1]. In the area of attachment of the valve cusp-like structure to the ventricular wall, there was a markedly thickened layer of elastic fibers but without their continuation into the cusp body, which was different from the normal aortic valve with characteristic thick layer of elastic fibers at the ventricular side of the valve cusps wall that extend from the thick elastic layer in the endocardium above the annulus fibrosus [11].

The abnormal accessory reverse-oriented tricuspid semilunar valve-like structure described in our case could remotely suggest subaortic membrane. This condition causes circumferential subaortic stenosis that ranges from a discrete subaortic membrane to a tunnel-type narrowing of the entire left ventricular outflow tract to stenosis associated with multilevel obstruction in Shone's complex [12]. Subaortic membrane is usually diagnosed early in childhood with clinical manifestation of aortic stenosis, although this lesion may be diagnosed also late in adulthood [13]. The histological structure of subaortic membranes is characterized by stack-like layers of fibroelastic tissue with underlying bundles of smooth muscle cells towards the subendocardial region [14], which is different from the observed histological composition of the above-described valve-like structures.

Accessory mitral valve leaflet is another lesion in the left ventricular outflow tract to be considered. This lesion leads to subvalvular aortic stenosis and usually also to severe aortic incompetence. In our case, semilunar valve-like structures were positioned on the muscular part of the outflow channel, which differed from the accessory mitral valve leaflet that outgoes from the membranous structure—the anterior mitral valve leaflet, and is connected to the anterolateral papillary muscle via a strand of chordal tissue [15]. Other pathologies that could produce left ventricular outflow obstruction account tumors, that is, myxoma [16] or a dynamic left ventricular outflow obstruction induced by other preexisting pathological conditions [17, 18].

In conclusion, the above-reported accessory reverse-oriented tricuspid semilunar valve-like structure is an unusual finding of an obstruction in the left ventricular outflow tract, to which we could not find an analogy in the available literature. The patient's history does not elucidate the pathogenesis of the lesion, whether it was a congenital or an acquired-during-life structure. Since no inflammatory cells, nor structures that could be considered remains of a reparatory process in the area were recorded, the second option is not likely.
